# Physical activity and somatic symptoms among hemodialysis patients: a multi-center study in Zhejiang, China

**DOI:** 10.1186/s12882-019-1652-z

**Published:** 2019-12-26

**Authors:** Xiaowei Lou, Yiwen Li, Huajuan Shen, Jin Juan, Qiang He

**Affiliations:** 1 The Second Clinical Medical College, Zhejiang Chinese Medical University, Hangzhou, Zhejiang 310014 People’s Republic of China; 2 Department of Nephrology, Zhejiang Provincial People’s Hospital, Hangzhou, Zhejiang 310014 People’s Republic of China; 3 Peoples’ Hospital of Hangzhou Medical College, Hangzhou, Zhejiang 310014 People’s Republic of China; 4 Chinese Medical Nephrology Key Laboratory of Zhejiang Province, Hangzhou, Zhejiang 310014 People’s Republic of China

**Keywords:** Chronic kidney disease patient, Medically unexplained symptoms, Somatic symptoms, Physical activity measures, Exercise therapy

## Abstract

**Background:**

Somatic symptoms are commonly reported by patients on maintenance hemodialysis. Based on evidence that exercise can improve psychological state among the general population, we aimed to evaluate the effects of physical activity on somatic symptoms specifically in this clinical population.

**Methods:**

This was a multicenter, cross-sectional study that included patients receiving hemodialysis treatment ≥3 times per week for > 3 months, aged 18 years or older, and who were willing to complete our study questionnaires and wear a pedometer; they were recruited from four hemodialysis centers in Zhejiang, China. Physical activity was quantified using pedometer data, with somatic symptoms quantified using the Symptom Checklist-90 (SCL-90). Hemodialysis information and blood laboratory tests were obtained from patients’ medical record. The score on the somatic dimension of the SCL-90 (S1-score) subdivided into tertiles for analysis: ≤1.17 (Q1), 1.17–1.58 (Q2) and ≥ 1.58 (Q3). A multivariate logistic regression analysis was performed to estimate the crude and adjusted odd ratios (ORs) and 95% confidence intervals (CIs) for the S1- somatic score according to the physical activity level during the last week. For this analysis, patients were stratified in a high and low exercise group using a cutoff of 3000 MET-min/week. Model 1 was adjusted for skinfold thickness of the triceps, upper arm circumference, grip strength, 5-m walking time, and 30-s sit-to-stand test. In model 2, we further adjusted for the leukocyte count, high-sensitivity C-reactive protein level, and albumin level.

**Results:**

After screening, 320 patients were enrolled into the study group (37.50% male, average age of 58.60 ± 14.2 years and mean average number of steps per day of 3725.92 ± 2663.47). The S1-score (1.51 ± 0.39) was significantly higher for patients than for the normal reference population (*P* < 0.001). As the S1-score increased, the average number of steps per day decreased, both on dialysis and non-dialysis days. Total physical activity, measured by pedometry, showed the best correlation to S1 scores (*r* = − 0.813; *P* < 0.01). The OR of a high S1-score was 1.97 [95% CI, 0.63–4.08] for patients in the low physical activity group.

**Conclusion:**

Higher S1 (somatic symptom) score was related to low physical activity among patients on maintenance hemodialysis.

## Background

End-stage renal disease (ESRD) is a leading cause of death in patients with chronic kidney disease (CKD). The rising prevalence of CKD has resulted in a significant increase in ESRD-related morbidity, worldwide [1]. Currently, maintenance hemodialysis (MHD) is the most widely used therapeutic approach for the clinical treatment of ESRD [2, 3]. While the continual development of hemodialysis technology has significantly prolonged the survival of patients on MHD, the quality of life of these patients remains low [4, 5].

Any illness or disease can produce important emotional, psychological and social responses that will impact perceived quality of life [6, 7]. Impairment in kidney function and urinary loss is not any different. The long course of the disease, combined with a sense of loss of body parts and the threat of death, as well as changes in diet, the high cost of dialysis and the development of arteriovenous fistula, can be sources of intense emotional and psychological discomfort [8]. Levy believes that the dependency on hemodialysis for survival places these patients in an unusual psychological state, and is often a source of severe depression [9, 10]. These experiences can lead to the development of somatic symptoms, such as sleep disorders, hand numbness and low back pain.

In the general population, low levels of physical activity are associated with somatic symptoms, such as sleep disturbances and depression, especially among elderly individuals [11–13]. Studies have shown that exercise can improve most psychological problems [14, 15], as well as relieve fatigue and improve physical symptoms [16]. It is speculated that increased physical activity may be an effective, non-drug, intervention to improve somatization symptoms. In small randomized controlled trials, increasing the daily physical activity of patients relieved symptoms of insomnia and restless leg syndrome [17–19], as well as improving symptoms of depression [20]. Among patients with depression, increasing the amount of daily physical activity has been associated to a higher rate of symptom remission and lower rate of relapse compared to patients treated with sertraline [21]. Low daily physical activity is a common problem among patients on MHD; however, the association between personal daily activity and the somatic symptoms in this clinical population has not been previously evaluated. Therefore, the aim of our study was to evaluate the prevalence of somatic symptoms among patients on MHD, and to assess the association between physical activity and somatic symptoms.

## Methods

### Study design, patient selection and statement of ethics

Four hemodialysis centers, from different cities in China (Hangzhou, Tiantai, Tongxiang, and Haining), participated in our multicenter prospective study. Prospective patients were those who had been receiving hemodialysis treatment for > 3 months, at least 3 times per week, were 18 years of age or older, and were willing to complete our study questionnaires and wear a pedometer. Excluded were patients who had a physical impairment, used a wheelchair, and/or had a severe cardiovascular or pulmonary disease limiting walking capacity, as well as those with a diagnosis of Alzheimer’s Disease, intellectual disability, illiteracy, or any other condition that would limit the patient’s ability to answer the study questionnaires.

**Fig. 1:**
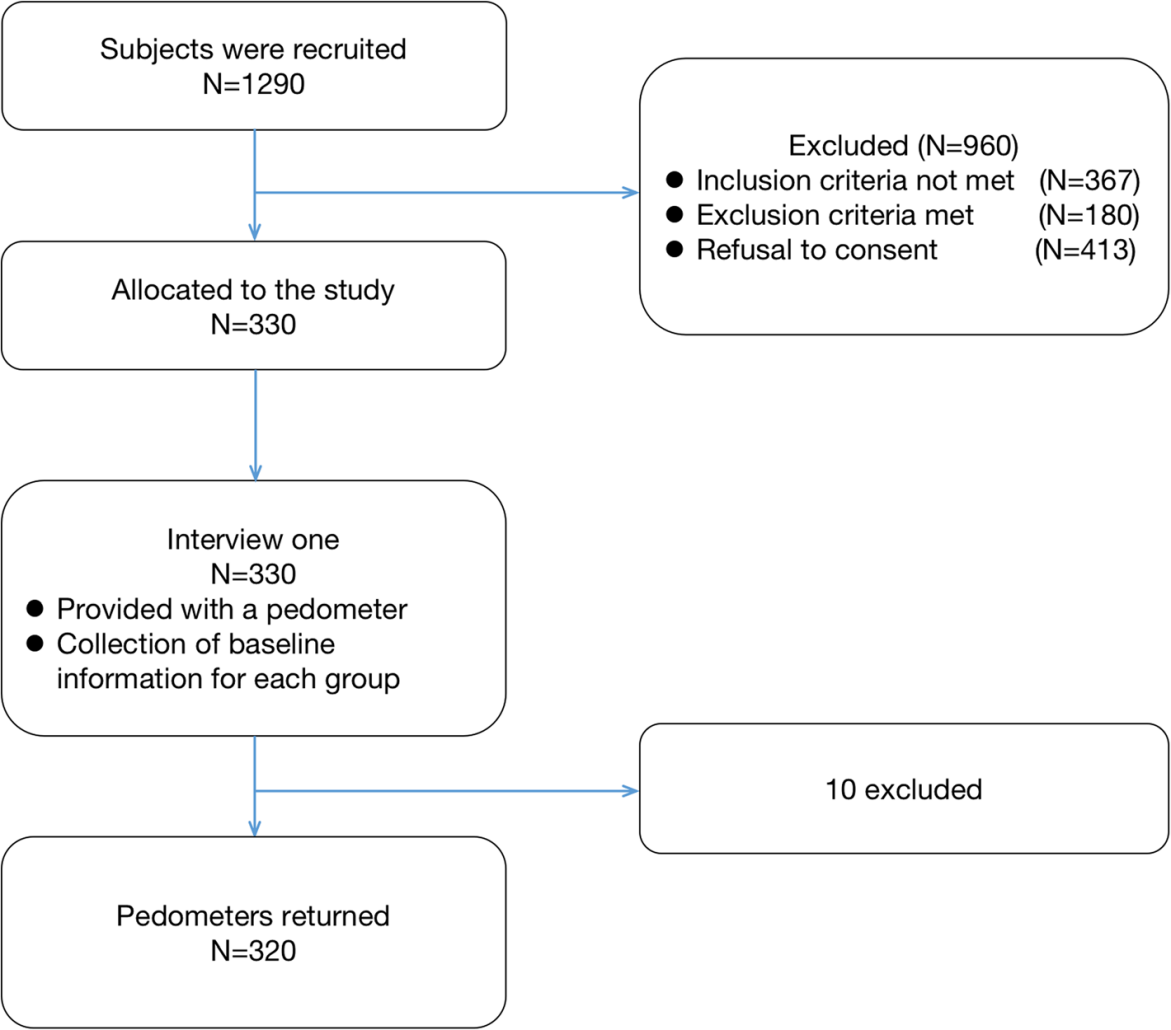
Flow chart of enrolment of patients on maintenance hemodialysis

The 1290 prospective patients identified across the 4 hemodialysis centers, 960 were excluded for the following reasons: 367 did not meet the inclusion criteria; 180 met one or more of the exclusion criteria; and 413 did not provide consent at the time of collecting baseline information. The remaining 330 patients were enrolled into the study. The flow chart of the overall study is shown in Fig. 1.

After enrollment, patients completed the SCL-90 symptom questionnaire, as well as the personal information study survey. Hemodialysis information and laboratory test results were obtained from each patient’s medical record. Pedometers were issued, and pedometer data collected over a 1-week period, as per previously published methods [22]. Patients completed the International Physical Activity Questionnaire (IPAQ; Chinese short version) when they returned their pedometer.

At the end-point of data collection, 10 patients were excluded due to missing baseline or pedometer, with the data from the remaining 320 patients entered into the analysis.

### Pedometer

The OMRON pedometer (HJ-328, Omron, Kyoto, Japan), which is an electronic motion sensor, was used in our study. The pedometer was worn around the waist to record any body movement. The effectiveness of the OMRON pedometer to measure physical activity has previously been proven in different populations [23, 24] and under different walking conditions [25–27]. The OMRON pedometer has been shown to provide a more reliable step count compared to other pedometers [28]. Previous studies [29] have shown that within a week, between-day differences were significant, but the differences appeared to be limited primarily to Sunday, and any 3-day combination (including those containing Sundays) could provide a sufficient estimate of the mean pedometer-determined steps/day. Considering the particularity of Sunday, patients in this study wore the pedometer for 7 days to provide a sufficient estimate.

The following two methods were used to improve adherence with wearing the pedometer. First, the pedometer was attached to a belt using a disposable plastic buckle and the belt was worn consistently for the 1-week recording period.

Second, all patients in the study received hemodialysis treatment three times a week, and pedometer use was confirmed at each MHD visit. On non-hemodialysis days, follow-up was conducted by video call.

### International Physical Activity Questionnaire (IPAQ; Chinese short version)

The IPAQ was developed by the World Health Organization, in 1998, to assess physical activity [30]. Two versions of the IPAQ are available: the long form (IPAQ-LF), which includes 31 questions, and the short form (IPAQ-SF), which includes 9 questions. Considering the burden already placed on patients undergoing MHD, we selected the IPAQ-SF. The total IPAQ score is converted to a metabolic equivalent (MET) minutes per day of physical activity.

### The symptom checklist-90 (scl-90)

The SCL-90 is a self-report questionnaire that evaluates the extent of symptoms on different factors. The SCL-90 is mainly used to quantify the psychological symptoms in patients with mental health disorders and those with physical diseases. The SCL-90 was first developed by combining the Cornell Medical Index [31] and the Hopkins Symptom Self-rating scale [32]. The SCL-90 was first presented by Derogatis et al. [33] in 1973. After several revisions, Derogatis et al. [34] published the revised SCL-90 (SCL-90-r), which is the version that is now principally used to screen for psychological symptoms. The version of the SCL-90-r used in mainland China was translated by Wang [35]. The reliability and validity of the translated SCL-90-r was confirmed by Jinhua et al. in a population of 1388 healthy adults in a multi-center and multi-area study, and is now the norm used to screen for psychological symptoms among Chinese populations. In our study, we used the short version of the SCL-90-R Chinese, translated by Wang.

The questionnaire consists of 90 questions, distributed across nine primary symptom dimensions [34], as follows: S1-Somatization (SOM), S2-Obsessive Compulsive (0-C), S3-Interpersonal Sensitivity (INT), S4-Depression (DEP), S5-Anxiety (ANX), S6-Hostility (HOS), S7-Phobic Anxiety (PHOB), S8-Paranoid Ideation (PAR), and S9-Psychoticism (PSY). The somatization dimension (S1) score, which reflects the level of distress caused by one’s perceived bodily dysfunction, is obtained by summing the score on the following 12 items of the SCL-90-r: 1, 4, 12, 27, 40, 42, 48, 49, 52, 53, 56 and 58. Complaint focus primarily on cardiovascular, gastrointestinal, and respiratory systems, with symptoms of headache, as well as pain and discomfort localized in the gross musculature being represented.

In addition to using S1 score, we calculated the rate of positive cases of psychological cases, defined as the number of person-positive cases normalized to the total number of patients enrolled. A person-positive case refers to a SCL-90r score greater than the norm reference. We used the normative SCL-90-r data for elderly individuals published by Chen and LI et al. [36], which was based on the scores of 555 (54.8% male) older individuals (average age, 68.1 ± 5.1 years) included in the mental health prevention and treatment network of Hangzhou city.

### Physical fitness assessment and laboratory test

We conducted a 2-h intake interview with each patient to gather detailed baseline information and conducted a physical fitness assessment. The following physical fitness factors were included in our analysis: thickness of triceps skinfold (TSF) and mid-arm muscle circumference (MAMC), measured using Holtain® skinfold calipers (Holtain Ltd., Crymych, UK) in accordance with the methods of auxological anthropometry [37]; grip strength, measured using the Jamar dynamometer (Sammons Preston, USA) [38]; the 5-m walking time, measured along a straight path using a stop watch; and the 30-s sit-to-stand test. All measures were performed twice, with at least a 5-min interval between repeat measurements, with the average of the two measures used in the analysis. The patient’s hemodialysis information, primary disease diagnosis and other laboratory test results were obtained from each patient’s medical chart.

### Statistical analyses

Data were reported as the mean and standard deviation for normally distributed continuous data, with the median and interquartile range (IQR) calculated for non-normally distributed continuous variables, and percentages for categorical data.

For analysis, the SCL-90 data was divided into 10 dimensions, with the 10th dimension discarded as per previous methods [33, 34]. The score in each dimension was compared to population norms using an independent *t*-test. The score on the first dimension (S1-somatic) was stratified into tertile scores (≤1.17 (Q1), 1.17–1.58 (Q2) and ≥ 1.58 (Q3), and differences in baseline characteristics between the three quartile groups (G1, G2 and G3, respectively) evaluated using an analysis of variance (ANOVA), Kruskal–Wallis test, or chi-squared test (χ2), as appropriate for the data type and distribution. Pearson’s correlation coefficient was calculated between baseline factors with a significant difference.

Finally, a multivariate logistic regression analysis was performed to estimate the crude and adjusted odd ratios (ORs), with the corresponding 95% confidence intervals (CIs), for the S1- somatic score according to the physical activity level during past week. For this analysis, patients were stratified in a high and low exercise group, using a cutoff of 3000 average steps/day. In the regression model, physical activity (high or low group) was the dependent variables and the somatic score (low, medium or high group) the independent variable. In model 1, we adjusted for thickness of the triceps skinfold, upper arm circumference, grip strength, 5-m walking time, and the 30-s sit-to-stand test, as covariates. In model 2, we further adjusted for the leukocyte count, high-sensitivity C-reactive protein level and albumin level as covariates. All three regression models (crude, 1 and 2) were used to evaluate the influence of high and low physical activity on S1-dimension scores.

Statistical analysis was performed using SPSS 20.0 (SPSS, Inc., Evanston, IL) and R × 64 3.6.0 (Development Core Team, New Zealand), with a 2-sided *P*-value < 0.05 indicative statistical significance.

## Results

Our analysis included 320 patients (mean age ± SD, 58.60 ± 14.2 years), 37.50% of whom were men, with 57.50% of participants having chronic glomerulonephritis, 27.50% diabetic nephropathy, and 3.44% hypertensive nephropathy. The physical fitness indices were significantly different between the three S1-tertile groups (all *P* < 0.001). As the S1 score increased, thickness of the triceps skin-fold, upper arm circumference, grip strength, and the number of 30-s sit-to-stand repetitions decreased, while the 5-m walk time increased (Table 1).

**Table 1:** Baseline characteristics of patients in each of the S1-somatic score tertile group (*n* = 320)

	Overall	Group one	Group two	Group three	*P* value
		(low; *n* = 109)	(medium; *n* = 114)	(high; *n* = 97)	
S1 score	1.51 ± 0.39	1.09 ± 0.06	1.40 ± 0.13	1.95 ± 0.30	
Age, years	58.60 ± 14.2	57.52 ± 16.54	56.90 ± 13.35	61.80 ± 19.11	0.89
Sex					
Male	120 (37.50%)	46 (42.20%)	40 (35.08%)	34 (35.05%)	0.76
Female	200 (62.50%)	63 (57.79%)	74 (64.91%)	63 (64.94%)	0.81
Dialysis duration, years	2.13 ± 1.19	1.89 ± 1.14	2.21 ± 1.23	1.82 ± 1.24	0.88
BMI, kg/m2	21.33 ± 5.12	20.46 ± 5.31	21.22 ± 5.43	22.41 ± 4.43	0.51
Original diseases					
Chronic glomerulonephritis	184 (57.50%)	58 (53.21%)	69 (60.53%)	57 (58.76%)	
Diabetic nephropathy	88 (27.50%)	30 (27.52%)	29 (25.44%)	29 (29.90%)	
Hypertensive nephropathy	11 (3.44%)	4 (3.67%)	4 (3.51%)	3 (3.09%)	
Other or unknown	37 (11.56%)	17 (15.60%)	12 (10.53%)	8 (8.25%)	
Physical fitness index					
TSF, mm	6.75 [5.00–9.00]	10.00 [9.00–12.88]	6.25 [6.00–7.13]	4.00 [4.00–4.38]	*P* < 0.001^#^
MAMC, mm	24.58 ± 3.06	27.85 ± 1.87	24.35 ± 0.63	21.19 ± 1.70	*P* < 0.001^#^
Grip strength, N	26.50 [20.60–34.25]	36.35 [33.15–38.93]	25.95 [24.10–27.73]	17.40 [10.60–19.30]	*P* < 0.001^#^
5-m walking time (s)	5.00 [4.07–6.00]	4.00 [4.00–4.25]	5.00 [5.00–5.31]	6.58 [6.00–9.83]	*P* < 0.001^#^
30 s Sit-to-stand test	16.50 [13.00–20.00]	20.50 [20.00–22.00]	16.00 [15.00–17.00]	11.50 [9.00–13.00]	*P* < 0.001^#^
Physical activity					
IPAQ-C (MET-min/week)	3251.43 ± 4000.49	5348.20 ± 4844.82	2798.31 ± 3583.50	1132.95 ± 1458.38	*P* < 0.001^#^
Pedometer (average steps per day)					
Total	3725.92 ± 2663.47	6564.83 ± 2426.82	3217.42 ± 579.50	1151.25 ± 661.75	*P* < 0.001^#^
Dialysis day	3218.08 ± 2264.80	5242.77 ± 2498.89	2865.43 ± 918.89	1370.08 ± 929.61	*P* < 0.001^#^
Non-dialysis day	4367.31 ± 3606.56	8151.58 ± 3158.02	3702.37 ± 1498.03	920.10 ± 809.94	*P* < 0.001^#^
UC, %	0.69 [0.64–0.74]	0.68 [0.63–0.72]	0.69 [0.66–0.74]	0.70 [0.67–0.76]	0.463
Laboratory indices					
iPTH, pg/ml	319.60 [154.75–591.75]	338.20 [162.68–628.75]	251.00 [148.00–454.25]	355.50 [155.00–595.25]	0.29
Ca, mmol/L	2.14 ± 0.25	2.15 ± 0.25	2.14 ± 0.25	2.14 ± 0.26	0.598
P, mmol/L	38.35 [36.10–40.40]	41.80 [40.00–43.08]	38.40 [36.70–39.03]	35.65 [33.25–36.50]	0.946
K, mmol/L	5.09 ± 0.95	5.01 ± 0.97	4.93 ± 0.85	5.36 ± 1.01	0.527
Mg, mmol/L	1.04 ± 0.15	1.02 ± 0.11	1.02 ± 0.14	1.08 ± 0.18	0.83
LC, 10^9/L	6.00 [4.78–6.99]	4.57 [3.85–5.00]	5.95 [5.45–6.52]	7.28 [6.52–8.17]	*P* < 0.001^#^
NC, 10^9/L	4.00 [3.28–5.00]	4.10 [3.53–5.15]	3.60 [3.00–4.45]	4.10 [3.53–5.38]	0.512
hsCRP, mg/L	1.50 [0.50–4.50]	0.50 [0.00–0.50]	1.40 [0.90–3.10]	6.00 [3.13–12.85]	*P* < 0.001^#^
HB, g/L	106.34 ± 14.40	107.89 ± 16.39	102.70 ± 11.75	108.88 ± 14.40	0.131
SI, μmol/L	11.80 [8.90–14.85]	12.65 [10.00–15.05]	11.20 [7.70–16.20]	11.60 [9.20–13.40]	0.203
Tfs, %	26.75 [18.53–34.50]	27.25 [21.85–36.40]	26.50 [15.85–35.60]	26.30 [22.60–31.80]	0.249
SF, μg/L	280.63 ± 257.34	293.78 ± 291.50	335.02 ± 265.10	199.57 ± 181.82	0.075
BPC, 10^9/L	176.29 ± 63.54	173.82 ± 57.04	166.60 ± 49.65	190.44 ± 81.79	0.265
Alb, g/L	38.31 ± 5.78	42.89 ± 4.54	38.16 ± 0.89	33.37 ± 6.25	*P* < 0.001^#^
TC, mmol/L	4.35 ± 1.15	4.37 ± 1.23	4.36 ± 1.01	4.32 ± 1.25	0.785
TG, mmol/L	2.25 ± 1.59	2.06 ± 1.55	2.06 ± 1.32	2.68 ± 1.88	0.254
LDL, mmol/L	2.35 ± 0.90	2.35 ± 0.77	2.49 ± 0.96	2.17 ± 0.94	0.192

Categorical data shown as count (percentage); continuous data, as mean ± standard or median [IQR] as appropriate. Groups are S1 score-specific: G1 (low): S1-somatic score 1.00–1.17, G2 (medium): S1-somatic score 1.25–1.58, G3 (high): S1-somatic score 1.67–2.83

*BMI* Body mass index, *TSF* Thickness of triceps skinfold, *MAMC* Midarm Muscle Circumference, *UC* Urea clearance, *iPTH* immunoreactive parathyroid hormone, *Ca* calcium, *P* Phosphorus, *K* Potassium Kalium, *Mg* Magnesium, *LC* Leukocyte count, *NC* Neutrophil count, *hsCRP* hypersensitive C-reactive protein, *HB* Hemoglobin, *SI* Serum iron, *Tfs* transferrin saturation, *SF* Serum ferritin, *BPC* blood platelet count, *Alb* Albumin, *TC* Total cholesterol, *TG* Triglyceride, *LDL* Low Density Lipoprotein

^#^ significant difference between those three groups

In terms of physical activity, the mean average steps per day was 3725.92 ± 2663.47. As the S1 scores increased, the average steps per day decreased, both on dialysis and non-dialysis days (*P* < 0.001): G1, 6564.83 ± 2426.82 steps; G2, 3217.42 ± 579.50 steps; G3, 1151.25 ± 661.75 steps (Table 1).

We report the Pearson’s rank-order of S1 scores and measured indices in Table 2, with all correlations being significant (*r* > 0.3; *P* < 0.05). The highest correlation was identified between the S1 score and pedometry measures (*r* = − 0.813; *P* < 0.01) and serum albumin level (*r* = − 0.807; *P* < 0.01).

**Table 2:** Correlations between the physical fitness and blood serum indices and S1 score

Index	Pearson Correlation
TSF	−.736^a^
MAMC	−.735^a^
Grip strength	−.660^a^
5-m walking time	.726^a^
Sit-to-stand test	−.366^a^
Average step number	−.813^a^
LC	.470^a^
hsCRP	.504^a^
Alb	−.807

Average step number a patient performed each day for a week in our study

*TSF* Thickness of triceps skinfold, *MAMC* Midarm Muscle Circumference, *LC* Leukocyte count, *hsCRP* hypersensitive C-reactive protein, *Alb* Albumin

^a^Correlation is significant at the 0.01 level (2-tailed). Statistical analysis was performed using the Pearson chi-squared test

The average score for each of the dimensions of the SCL-90 (short form) are reported in Table 3. All scores were significantly higher among our patient group than the norm reference group (all *P* > 0.05). The highest proportion of expressed symptoms were somatic (53.44%), followed by phobia (33.13%) and psychosis (30.31%).

**Table 3:** Score on the nine dimensions of the SCL-90 between patients and the norm reference

Group	Patients	Norm	Positive rate	*P*
Clinical cases	320	555		
S1-Somatic	1.46 ± 0.40	1.37 ± 0.48	53.44%	< 0.001
S2-Obsessive	1.39 ± 0.38	1.62 ± 0.58	18.75%	< 0.001
S3-Interpersonal sensitive	1.22 ± 0.34	1.65 ± 0.51	10.63%	< 0.001
S4-Depression	1.39 ± 0.46	1.50 ± 0.59	24.69%	0.001
S5-Anxiety	1.30 ± 0.38	1.39 ± 0.43	29.06%	0.001
S6-Hostility	1.19 ± 0.29	1.48 ± 0.56	12.81%	< 0.001
S7-Phobia	1.20 ± 0.29	1.23 ± 0.41	33.13%	0.063
S8-Paranoia	1.15 ± 0.23	1.43 ± 0.57	9.69%	< 0.001
S9-Psychosis	1.21 ± 0.25	1.29 ± 0.43	30.31%	< 0.001

Norm: one data series from Zhejiang, the effective number was 555, including 304 males (54 8%) and 251 females (45.2%), with an average age of (68.1 ± 5.1) years

Positive rate = Above normal number/ total number

P (comparisons among patients and norm)

To evaluate the association between physical activity and the S1-score at baseline, patients were dichotomized into a high and a low physical activity group, using a cutoff of 3000 MET-min/week. The crude OR of a high S1-score was significantly higher in patients with lower physical activity (OR, 2.08 [95% CI, 1.01–4.29] for the lowest versus highest tertile S1-group, *P* < 0.05). This relationship was maintained for the adjusted OR for model 1 and model 2; Table 4).

**Table 4:** Crude and Adjusted Odd Ratios for S1- Somatic Score According to physical activity

	Odds ratio^a^	*P*-value
Crude Model	2.08(1.01–4.29)	0.038
Model 1	2.02(0.98–4.17)	0.047
Model 2	1.97 (0.63–4.08)	0.048

Model 1: adjusted for Physical fitness indices, including thickness of triceps skinfold, upper arm circumference, grip strength, 5-m walking time, and 30-s Sit-to-stand test

Model 2: model 1 + leukocyte count, high-sensitivity C-reactive protein level, and albumin level

^a^values shown are odd ratios (95% confidence interval)

## Discussion

Although survival of patients on MHD has been prolonged, this has not translated to an improvement in quality of life [39]. Somatic discomfort, which includes fatigue, back pain, leg pain, and sore throat, is the leading cause of poor quality of life outcomes in this clinical population [40]. In many cases, these somatic manifestations arise from psychological, rather than physiological, causes, and are referred to as medically unexplained symptoms [41]. Various factors can affect the psychological state of patients on MHD, including the high medical costs, monotonous periods of hospitalization, lack of care from relatives, and long-term suffering, among other factors [42]. The latest international research suggests that low physical activity can lead to symptoms, such as depression [43] and physical discomfort [44], among patients on hemodialysis. Exercise therapy plays an important role in the clinical management of patients on MHD, improving physical disability [45] and long-term survival [46], as well as relieving symptoms of fatigue and insomnia [47]. An international multicenter study reported that exercise patterns [48] among patients on MHD could be standardized across different regions and countries, regardless of language barriers. Therefore, we believe that our study, performed in China, is a first step toward that goal. Our specific interest in exploring the possible association between physical activity and somatic symptoms among patients on MHD was based on evidence that exercise exerts a positive effect in healthy individuals.

Our findings on the SCL-90 confirm that patients on MHD have an overall poorer psychological status than the general population, which is consistent with a previous report [49]. Of the 9 dimension scores of the SCL-90, we identified somatic symptoms as being the most prevalent psychological issue among this clinical population. A previous study has demonstrate that severe somatization can negatively impact quality of life [50]. We also demonstrate that patients in the highest tertile of S1-scores had a higher inflammation index, lower nutritional status index, as well as lower levels of physical exercise and daily activities. All these indices were moderately correlated with S1-scores. On regression analysis, patients with low physical activity (average daily steps < 4000 steps, 3000 MET-min/week [51]) were 2.08 times more likely report somatization symptoms than those with high physical activity. After adjusting for other factors (inflammation, nutritional, and physical fitness status), the probability of higher somatization with lower physical activity level remained high at 1.97, confirming a certain correlation between low exercise volume and somatization symptoms among patients on maintenance hemodialysis. Since our study used a cross-sectional design, we cannot directly determine whether low physical activity is the cause of somatization. However, previous studies have shown that some several musculoskeletal symptoms, such as pain and fatigue, and depressive symptoms may be caused by physical inactivity and malnutrition, both among patients on hemodialysis [52, 53] and in healthy elderly individuals [54, 55]. We therefore speculate that low physical activity may be one of the factors contributing to somatic symptoms. However, the exact nature of the interactions between somatic symptoms and physical activity needs further evaluation. It is therefore possible that patient education to encourage physical activity and reduce sedentary lifestyle could reduce somatization discomfort and improve the quality of life of patients on maintenance hemodialysis.

### Limitations of the study

The limitations of our study need to be acknowledged in the interpretation of our findings. First, our study relied on a single pedometry measure to quantify physical activity, and an accelerometer, considered to be the gold standard for measuring daily physical activity, was not used to the size and cost. We do note that 413 eligible patients refused to participate, largely due to the inconvenience of wearing a pedometer for a 1-week period of recording. There was no significant difference in the distribution of patients who refused to participate compared to our study group.

To improve recruitment, as possible, the study should be expanded nationwide and even internationally. The cross-sectional design did not allow for causal inferences between physical activity level and S1 scores. Furthermore, we used the SCL-90 and IPAQ questionnaires owing to their practicality of use. However, issues of reliability bias have been raised with these questionnaires and thus, other mental health tests and physical activity measurements having higher reliability could be used in the future. We also note that the blood tests and measures of physical fitness collected were limited and, thus, no conclusions can be drawn regarding the mechanism underlying the interaction between physical activity and somatic discomfort symptoms. In the next phase of the study, we will be including randomization and an exercise intervention, as well as expanding the range of laboratory measures includes to explore the signaling pathways involved in the association between physical activity and somatization in this clinical population.

## Conclusion

In summary, our study confirms a positive effect of daily physical activity in reducing somatization symptoms among patients undergoing maintenance hemodialysis. Reducing patients’ sedentary lifestyle and promoting exercise might be beneficial in improving quality of life in this clinical population.

## Data Availability

All data supporting the study are presented in the manuscript and available on a request to the corresponding authors of this manuscript, Qiang He.

## Abbreviations

BMI: Body mass index
Ca: Calcium
CKD: Chronic kidney disease
ESRD: End-stage renal disease
Hb: Hemoglobin
hsCRP: High-sensitivity C-reactive protein
IPAQ-C: International Physical Activity Questionnaire
IPAQ-SF: International Physical Activity Questionnaire short version
iPTH: Intact paratahyroid hormone
IQR: Interquartile range
K: Potassium
LDL: Low density lipoprotein cholesterol
Mg: Magnesium
MHD: Maintenance hemodialysis
OR: Odds ratio
P: Phosphorus
PA: Physical activity
SCL-90: Symptom checklist- 90
SD: Standard deviation
